# Syndrome coronarien aigue suite à une injection accidentelle intraveineuse de Bupivacaine

**DOI:** 10.11604/pamj.2015.22.144.7136

**Published:** 2015-10-15

**Authors:** Siriman Abdoulaye Koita, Najib Bouhabba, Fjouji Salaheddine, Mustapha Bensghir, Haimeur Charki

**Affiliations:** 1 Pôle Anesthésie Réanimation Hôpital Militaire Med V Rabat, Faculté de Médecine et de Pharmacie de Rabat, Université Souissi Med V, Rabat, Maroc

**Keywords:** Rachianesthésie, sédation, erreur médicamenteuse, injection intraveineuse de bupivacaine, tachycardie ventriculaire, spinal anesthesia, sedation, medication error, intravenous injection of bupivacaine, ventricular tachycardia

## Abstract

Les erreurs médicamenteuses constituent une complication très redoutable en anesthésie. La fréquence des erreurs médicamenteuses par administration serait de l'ordre 1/10 000 à 1/1000. Nous rapportons un cas d'injection accidentelle de bupivacaine en intraveineuse directe. Il s'agissait d'un jeune patient classé ASA I qui était prévu pour cure chirurgicale d'une hydrocèle unilatérale sous rachianesthésie. Une injection de 12,5 mg de bupivacaine et 25 µg de fentanyl étaient injectés après un reflux net du liquide céphalorachidien sans incident. Après installation de la rachianesthésie, le début de l'intervention était autorisé sans incidents. Une sédation de confort était décidée. Juste après apparaissait des troubles de rythme à type tachycardie ventriculaire avec une élévation de taux de troponines en post opératoire. L’évolution était marquée par une normalisation de la troponine au troisième jour post opératoire et sorti de l'hôpital au cinquième jour.

## Introduction

Il s'agissait d'un patient âgé de 31 qui était prévu pour cure chirurgicale d'une hydrocèle unilatérale. Lors de la consultation pré anesthésique, on notait une intervention pour fracture de la jambe sous rachianesthésie sans incidents et aucun facteur de risque cardiovasculaire. Au terme de cette consultation le patient était classé ASA I et Mallampati I et une rachianesthésie, comme technique anesthésique, était décidée. Après admission au bloc opératoire, un monitorage standard incluant la pression non invasive (PNI), l’électrocardiogramme (ECG) et la saturation pulsée en oxygène (SPO2). Les paramètres de départ affichaient une PNI à 135/70 mmHg, une fréquence cardiaque (FC) à 85 batt/mn et une SPO2 à 100% à l'air ambiant. Une voie veineuse périphérique (18G) était prise et un remplissage par du sérum salé 0,9% (250ml) était démarré puis le patient était mis en position pour la rachianesthésie. Une ponction de l'espace inter épineux L4-L5 était faite permettant un reflux net du liquide céphalorachidien. Un mélange de 12,5 mg de bupivacaine et de 25 ‘g de fentanyl était injecté puis le patient était installé en décubitus dorsal. Aucune modification de la PNI et/ou de la FC n’était notée. Une sédation de confort était décidée (midazolam). Juste après, le patient avait présenté une sensation d'oppression thoracique avec des troubles neurologique à type de confusion et de dysarthrie. Le tracé ECG avait montré des salves d'extrasystole ventriculaire, la PNI était à 75/40 mmHg et la SPO2 était normale. Suite à ces troubles, une conversion en anesthésie générale était décidée. Une dose de 60 mg de lidocaine était administrée permettant la régression des troubles de rythme. A la fin de l'intervention qui avait duré 1h30 minutes, le patient était extubé après un réveil complet sans anomalies neurologiques ou rythmiques. La vérification du plateau des drogues n'avait objectivé aucune seringue de midazolam d'une part et d'autre part aucun flacon vide de midazolam n'a été retrouvé dans la salle. Une erreur médicamenteuse avec injection de la bupivacaine était très probable. Le bilan postopératoire avait montré une troponine positive à 1,530 ‘g /l (25 fois la normal) au premier jour puis 0,322 au troisième jour avec une négativation au sixième jour. L'ionogramme sanguin n'avait pas montré de trouble hydro électrolytique. L'ECG et l’échocardiographie trans thoracique étaient sans particularités. Un screening pour recherche toxicologique fait sur les prélèvements sanguins était revenu négatif aux benzodiazepines et positif à la mepivacaine dont la bupivacaine est un dérivé. Le diagnostic d'une erreur médicamenteuse par injection accidentelle de la bupivacaine à la place de midazolam était retenu. Le suivi ultérieur du patient était sans particularités. Le patient était déclaré sortant au cinquième jour post opératoire.

## Patient et observation

Il s'agissait d'un patient âgé de 31 qui était prévu pour cure chirurgicale d'une hydrocèle unilatérale. Lors de la consultation pré anesthésique, on notait une intervention pour fracture de la jambe sous rachianesthésie sans incidents et aucun facteur de risque cardiovasculaire. Au terme de cette consultation le patient était classé ASA I et Mallampati I et une rachianesthésie, comme technique anesthésique, était décidée. Après admission au bloc opératoire, un monitorage standard incluant la pression non invasive (PNI), l’électrocardiogramme (ECG) et la saturation pulsée en oxygène (SPO2). Les paramètres de départ affichaient une PNI à 135/70 mmHg, une fréquence cardiaque (FC) à 85 batt/mn et une SPO2 à 100% à l'air ambiant. Une voie veineuse périphérique (18G) était prise et un remplissage par du sérum salé 0,9% (250ml) était démarré puis le patient était mis en position pour la rachianesthésie. Une ponction de l'espace inter épineux L4-L5 était faite permettant un reflux net du liquide céphalorachidien. Un mélange de 12,5 mg de bupivacaine et de 25 ‘g de fentanyl était injecté puis le patient était installé en décubitus dorsal. Aucune modification de la PNI et/ou de la FC n’était notée. Une sédation de confort était décidée (midazolam). Juste après, le patient avait présenté une sensation d'oppression thoracique avec des troubles neurologique à type de confusion et de dysarthrie. Le tracé ECG avait montré des salves d'extrasystole ventriculaire, la PNI était à 75/40 mmHg et la SPO2 était normale. Suite à ces troubles, une conversion en anesthésie générale était décidée. Une dose de 60 mg de lidocaine était administrée permettant la régression des troubles de rythme. A la fin de l'intervention qui avait duré 1h30 minutes, le patient était extubé après un réveil complet sans anomalies neurologiques ou rythmiques. La vérification du plateau des drogues n'avait objectivé aucune seringue de midazolam d'une part et d'autre part aucun flacon vide de midazolam n'a été retrouvé dans la salle. Une erreur médicamenteuse avec injection de la bupivacaine était très probable. Le bilan postopératoire avait montré une troponine positive à 1,530 µg /l (25 fois la normal) au premier jour puis 0,322 au troisième jour avec une négativation au sixième jour. L'ionogramme sanguin n'avait pas montré de trouble hydro électrolytique. L'ECG et l’échocardiographie trans thoracique étaient sans particularités. Un screening pour recherche toxicologique fait sur les prélèvements sanguins était revenu négatif aux benzodiazepines et positif à la mepivacaine dont la bupivacaine est un dérivé. Le diagnostic d'une erreur médicamenteuse par injection accidentelle de la bupivacaine à la place de midazolam était retenu. Le suivi ultérieur du patient était sans particularités. Le patient était déclaré sortant au cinquième jour post opératoire.

## Discussion

Les erreurs médicamenteuses sont fréquentes et présentes à toutes les étapes du processus thérapeutique. En anesthésie, les études publiées mettent en évidence qu'une erreur médicamenteuse survient de 1 fois sur 900 à 1 fois sur 1/274 anesthésies [1, 2]. Cependant, ce chiffre est vraisemblablement sous-estimé car issu de déclarations volontaires, méthode peu adaptée à l'identification des erreurs médicamenteuses. En effet, il a été montré que la fréquence des erreurs observées pourrait être 400 fois supérieure à celle des erreurs déclarées [3]. Les des séries historiques rapportent des incidences extrêmes allant de 1/5 à 1/8000 [4]. Pour Webster et al, deux patients par anesthésiste seraient victimes d'effets indésirables liés à l'EM durant 30 ans d'exercice [5]. Fastinget al dans une enquête norvégienne qui concernait 55 426 anesthésies pendant une période de 36 mois, trouvaient une incidence des EM à 1/900. Webster et al, dans une autre étude prospective en Nouvelle-Zélande à propos de 10 806 anesthésies montraient une incidence des EM à 1/133 [5]. Une enquête prospective au niveau des CHU Marocains sur les EM en anesthésie par M. Amor et al relevait une incidence de 1/575 [6], rejoignant les résultats de l’étude rétrospective de Yamamoto et al réalisée en 2008, avec une incidence de 1/571 [7]. Tous ces résultats sont certainement sous-estimés puisque toutes les données publiées étaient issues de déclarations volontaires des praticiens et donc subjectives. En effet, l’étude de Barker et al qui a la particularité d'avoir été dirigée par des enquêteurs étrangers aux équipes sondées; présente un taux d'erreur très élevé [8] par rapport à toutes les autres publications dont le taux d'EM reste inférieur à 1% quel que soit le type d’étude conduite. Les erreurs médicamenteuses relevées en anesthésie concernent principalement par ordre de fréquence décroissante i) les seringues et les ampoules (50%); ii) les dispositifs médicaux d'administration (26%); iii) la voie d'administration (14%). Les erreurs relatives aux seringues et ampoules relèvent essentiellement dans 62% des cas d'une confusion de spécialité et dans 11%, d'une erreur de concentration du médicament. Lors de confusion de spécialités, l'erreur survient dans 55% des cas au moment de l'administration (erreur de seringue), et dans 45% pendant la reconstitution (erreur de spécialité, erreur d’étiquetage) [4]. Si on estime que 5 médicaments sont administrés en moyenne par anesthésie le risque de survenu d'erreur serait proportionnel au nombre de médicaments administrés. La fréquence des erreurs médicamenteuses par administration serait de l'ordre de 1 fois sur 10 000 à 1 fois sur 1 000 [9]. Ce qui explique l'incidence élevée de ces erreurs en anesthésie générale par rapport l'anesthésie locorégionale plus particulièrement en rachianesthésie. Dans la série de Amoret al, le manque d'expérience des praticiens semble être un facteur déterminant dans la survenue de certains types d'erreur médicamenteuse [6]. L'explication peut se trouver dans le manque de familiarisation des jeunes anesthésistes par rapport aux nomenclatures des médicaments et surtout par rapport à la complexité des modalités de dilution des substances. L’étiquetage des seringues se fait de façon manuscrite, celui des ampoules dépend des choix de l'industrie pharmaceutique locale et n'obéit à aucune réglé de standardisation. Cela est à la base de la survenue des erreurs de substitution. Ces pratiques ne répondent pas aux exigences des recommandations émises [9, 10].

En milieu pédiatrique le risque de survenu serait plus élevé que chez l'adulte. On avance une incidence de 1/405 EM en anesthésie pédiatrique [6]. Contrairement à l'adulte, la fréquence des erreurs de reconstitution (dilution) est aussi importante que celle des erreurs d'administration. Cela est facilement expliqué par le fait que les doses pédiatriques, calculées selon la masse corporelle, requièrent fréquemment des dilutions complexes des substances. Malgré l'augmentation de l'intérêt accordé à la prévention de l'EM à travers le monde et les nombreuses recommandations publiées par la plupart des sociétés savantes, il existe encore de grandes lacunes à combler concernant ce sujet.

Une réflexion pérenne doit être mené sur les moyens à mettre en oeuvre pour prévenir les erreurs médicamenteuses. Cette réflexion doit aboutir à la mise en oeuvre de mesures de prévention spécifiques, communes à l'ensemble de la structure et formalisées par écrit. Les actions mises en place doivent être réévaluées régulièrement. A cette fin, les événements médicamenteux indésirables évitables, avérés ou potentiels doivent pouvoir être déclarés, notamment à la commission du médicament et des dispositifs médicaux stériles, et faire l'objet d'une analyse détaillée. L'ensemble de la démarche conduite par la structure, doit être documentée, et les preuves de son existence apportées.

Les erreurs les plus fréquemment rencontrées sont celles des seringues et des ampoules, les erreurs de dilution; la voie d'administration. La première mesure est de rendre la déclaration des EM obligatoire. Cela permettra de publier des incidences réellement conforment à l'ampleur du problème. Cela pourrait mener à une prise de conscience chez les praticiens d'une part et chez les décideurs d'autre part aboutissant à la mise en place et l'application des mesures déjà prises.

Le meilleur moyen de lutter contre ce problème est la prévention, Nous recommandons des mesures préventives simples qui nous semblent essentielles et réalisables dans nos structures. La mise à disposition de plateaux d'anesthésié optimisé afin de permettre aux anesthésistes de bénéficier d'un espace de travail bien ordonné et le plus aseptique possible; La mise à disposition de seringues pré remplies pour les médicaments les plus couramment utilisés en anesthésie; Dans les erreurs de substitution il faut une attention particulière aux étiquetages. Le personnel doit accorder une importance capitale à la lecture attentive des informations notées sur l’étiquette. La mise à disposition d’étiquettes lisibles et conformes au code couleur international; l'application d'un système combinant non seulement l'utilisation de codes-barres, mais également la mise à disposition de seringues pré remplies, correctement étiquetées et rangées sur des plateaux d'anesthésie semble avoir porté fruit. Par ces mesures préventives, Haller G et al arrivaient à réduire l'incidence des EM dans leur centre [11]. Donc les seringues doivent être systématiquement étiquetées de maniéré lisible sans masquer leurs graduations. Ces étiquettes doivent mentionner la dénomination commune internationale du médicament avec un emplacement libre réservée à la mention de la concentration du médicament. Les seringues préparées doivent être rangées dans un plateau selon un plan prédéfini commun à la structure. La Prévention des erreurs de dilution repose sur la rédaction et l'application de protocoles de préparation des médicaments. Ces protocoles doivent préciser les modalités de reconstitution du médicament, la concentration du médicament (exprimée en mg/mL,UI/mL), le volume à préparer ainsi que celui de la seringue utilisée.

Une attention particulière doit être de mise lors d'une anesthésie pédiatrique. Une extrême vigilance est préconisée de la part des praticiens depuis le choix du produit jusqu’à son administration. Le choix du produit doit être nominal, une dilution exacte selon un protocole pré établit de chaque médicament. Le poids de l'enfant doit être toujours mentionné de façon précise sur le dossier médical. Chaque médicament doit être reconstitué et étiqueté au cours d'une seule séquence de préparation, sans interruption ni changement de lieu et par la même personne. Dans notre cas, Le diagnostic d'une erreur médicamenteuse par injection accidentelle de la bupivacaine à la place de midazolam était retenu suite à une erreur d'identification de l'ampoule. L'erreur médicamenteuse était classée comme grave vu la survenue d'une tachycardie ventriculaire. Les suites étaient simples sans séquelles.

## Conclusion

L'erreur médicamenteuse constitue un problème grave avec des conséquences parfois vitales en anesthésie. Malgré de nombreuses mesures entreprises en matière de prévention, son incidence reste élevée. La simple vigilance, la standardisation des protocoles d'anesthésie, le respect des recommandations et la prudence avant toute administration de médicament représentent les clés de la prévention.
